# S113R mutation in SLC33A1 leads to neurodegeneration and augmented BMP signaling in a mouse model

**DOI:** 10.1242/dmm.026880

**Published:** 2017-01-01

**Authors:** Pingting Liu, Baichun Jiang, Jian Ma, Pengfei Lin, Yinshuai Zhang, Changshun Shao, Wenjie Sun, Yaoqin Gong

**Affiliations:** 1 The Key Laboratory of Experimental Teratology, Ministry of Education and Department of Genetics, Shandong University School of Medicine, Jinan, Shandong 250012, China; 2 Laboratory of Neuromuscular Disorders and Department of Neurology, Qilu Hospital, Shandong University, Jinan, Shandong 250012, China

**Keywords:** SLC33A1, Bone morphogenetic protein (BMP), Knock-in mouse model, Neurodegeneration, Hereditary spastic paraplegia

## Abstract

The S113R mutation (c.339T>G) (MIM #603690.0001) in SLC33A1 (MIM #603690), an ER membrane acetyl-CoA transporter, has been previously identified in individuals with hereditary spastic paraplegia type 42 (SPG42; MIM #612539). SLC33A1 has also been shown to inhibit the bone morphogenetic protein (BMP) signaling pathway in zebrafish. To better understand the function of SLC33A1, we generated and characterized *Slc33a1^S113R^* knock-in mice. Homozygous *Slc33a1^S113R^* mutant mice were embryonic lethal, whereas heterozygous *Slc33a1* mutant mice (*Slc33a1^wt/mut^*) exhibited behavioral abnormalities and central neurodegeneration, which is consistent with hereditary spastic paraplegia (HSP) phenotypes. Importantly, we found an upregulation of BMP signaling in the nervous system and mouse embryonic fibroblasts of *Slc33a1^wt/mut^* mice. Using a sciatic nerve crush injury model *in vivo* and dorsal root ganglion (DRG) culture *in vitro* we showed that injury-induced axonal regeneration in *Slc33a1^wt/mut^* mice was accelerated and mediated by upregulated BMP signaling. Exogenous addition of BMP signaling antagonist, noggin, could efficiently alleviate the accelerated injury-induced axonal regrowth. These results indicate that SLC33A1 can negatively regulate BMP signaling in mice, further supporting the notion that upregulation of BMP signaling is a common mechanism of a subset of hereditary spastic paraplegias.

## Abstract (precis)

**Summary:** *Slc33a1^wt/mut^* knock-in mice with a S113R mutation and exhibiting hereditary spastic paraplegia-related phenotypes show that SLC33A1 negatively regulates BMP signaling and axonal regeneration.

## INTRODUCTION

Hereditary spastic paraplegia (HSP) is a clinically and genetically heterogeneous group of neurodegenerative disorders in which progressive lower extremity weakness and spasticity are the predominant clinical symptoms. HSP is usually caused by developmental failure or distal degeneration of motor axons in the corticospinal tract. To date, at least 72 different spastic gait disease loci and 59 corresponding spastic paraplegia genes (SPGs) have been identified. Functional studies of various HSP genes have revealed several cellular pathways that are dysregulated in HSP, including intracellular trafficking, mitochondrial function, lipid metabolism, myelination, BMP signaling and axonal transport (Blackstone et al., 2011; Blackstone, 2012; Fink, 2013; Klebe et al., 2015). However, the exact mechanisms underlying the pathogenesis of HSP are still unknown.

We have previously demonstrated that *SLC33A1* p.Ser113Arg (S113R) (c.339T>G) (MIM# 603690.0001) mutation causes autosomal-dominant spastic paraplegia type 42 (SPG42, MIM#612539) in humans (Lin et al., 2008). However, the proposition of SLC33A1^S113R^ as the causative mutation was challenged by the reports that no *SLC33A1* mutation has been detected in other HSP cases and homozygous mutations in *SLC33A1* cause a lethal recessive disorder with the phenotypes of congenital cataracts, hearing loss, and low serum copper and ceruloplasmin (Schlipf et al., 2010; Huppke et al., 2012). Because animal models often provide crucial evidence for a possible pathogenic role of a gene in question, we resorted to zebrafish and mouse models to determine whether the *SLC33A1^S113R^* mutation could cause HSP. We previously reported a significant elevation of BMP signaling in *slc33a1* knockdown zebrafish and in fibroblasts derived from an individual with SPG42 (Mao et al., 2015), indicating that *Slc33a1^S113R^* mutation likely causes HSP via affecting BMP signaling. We here present the results obtained from *Slc33a1^S113R^* knock-in mice. We found that the mutant mice developed behavioral abnormalities and central neurodegeneration consistent with pure HSP phenotypes. Moreover, upregulation of BMP signaling was found in the nervous system of *Slc33a1^S113R^* knock-in mice. Experiments using a mouse sciatic nerve crush injury model *in vivo* and DRG culture *in vitro* showed that SLC33A1 can negatively regulate BMP signaling, further supporting that upregulation of BMP signaling is a common mechanism of a subset of HSPs.

## RESULTS

### Homozygous S113R mutation in SLC33A1 led to developmental arrest

**Fig. 1.**
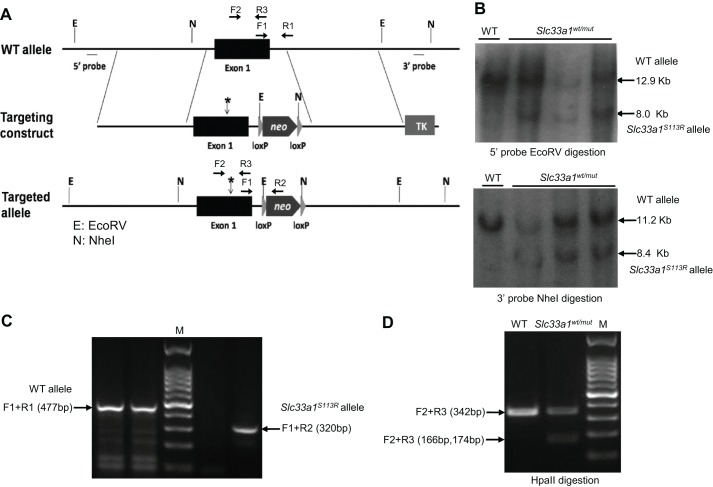
**Generation of *Slc33a1^S113R^* knock-in mice.** (A) The targeting strategy for the *Slc33a1* genomic locus. * indicates the S113R mutation. (B) Southern blotting analysis of the *Slc33a1* locus using genomic DNA isolated from WT and *Slc33a1^wt/mut^* mice. Samples were digested with either *Eco*RV (top panel) or *Nhe*I (bottom panel), and fragments were detected with 5′ probe or 3′ probe. (C) PCR-based identification of WT and *Slc33a1^wt/mut^* mice. PCR with *Slc33a1*-specific (F1 and R1) and *neo*-specific (R2) primers produced two independent products of 477 bp (F1+R1) and 320 bp (F1+R2) for the WT allele and *Slc33a1^S113R^* allele, respectively. (D) PCR-RFLP-based identification of WT and *Slc33a1^wt/mut^* mice. PCR products were digested with *Hpa*II. For WT mice, the digestion products yield only one band (342 bp), and for the *Slc33a1^wt/mut^* mice, the products contain three bands (342, 174 and 166 bp).

To examine the pathogenic effects of *SLC33A1^S113R^* mutation, we generated *Slc33a1^S113R^* knock-in mutant mice (Fig. 1). Intercross of *Slc33a1^S113R^* heterozygotes (*Slc33a1^wt/mut^*) produced 79 postnatal pups. Although there was a 2:1 ratio of heterozygotes to wild-type (WT) mice, no homozygous mutant pups were found, indicating that homozygotes for *Slc33a1^S113R^* are not viable.

**Fig. 2.**
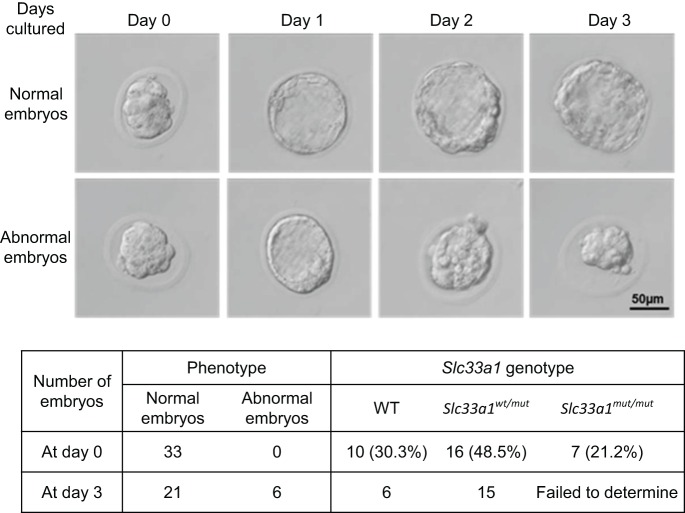
***Slc33a1^mut/mut^* embryos fail to survive *in vitro*.** Embryos harvested at E2.5 from heterozygous mutant intercrossed mice were cultured and imaged for four consecutive days. Upper panels: pictures of typical abnormal and normal embryos are shown. Lower panel: table summarizing the phenotype and genotype of all embryos cultured.

To determine at which developmental stage the homozygous mutant embryos die, embryos at embryonic day (E)7.5 and E18.5 from heterozygote intercrosses were isolated and genotyped by PCR. Homozygous mutant embryos were detected at neither stage, indicating that *Slc33a1^mut/mut^* embryos die prior to E7.5. In addition, all of the E7.5 implantation sites seemed normal, suggesting that the mutant embryos died prior to implantation and thus did not induce a decidual swelling in the uterus. We next harvested E3.5 embryos from heterozygote intercrosses and found that 5.7% of the blastocysts were *Slc33a1^mut/mut^*, far fewer than expected 25%, indicating that SLC33A1 function is required for blastocyst formation. However, E2.5 *Slc33a1^mut/mut^* blastocysts flushed from the oviduct were recovered at the expected Mendelian ratio (Table 1), indicating that most *Slc33a1^mut/mut^* embryos perish between E2.5 and E3.5. When the E2.5 embryos recovered from the oviducts were cultured, the *Slc33a1^mut/mut^* embryos showed great growth arrest (Fig. 2), further supporting the notion that *Slc33a1^S113R^* homozygous mutation is incompatible with pre-implantation development.

**Table 1.**
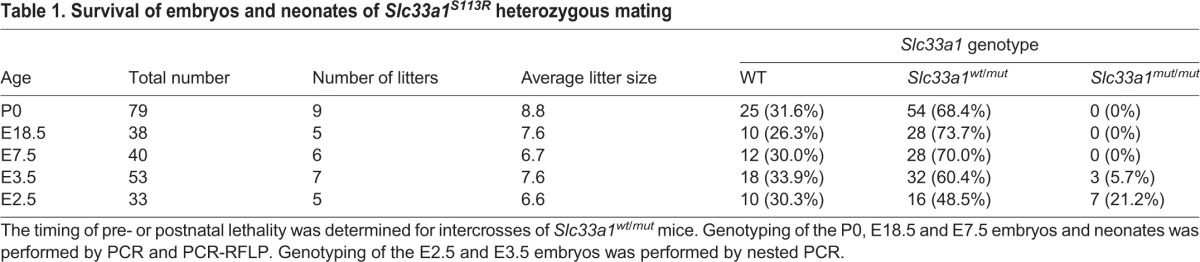
**Survival of embryos and neonates of *Slc33a1^S113R^* heterozygous mating**

### Motor movement of *Slc33a1^wt/mut^* mice

**Fig. 3.**
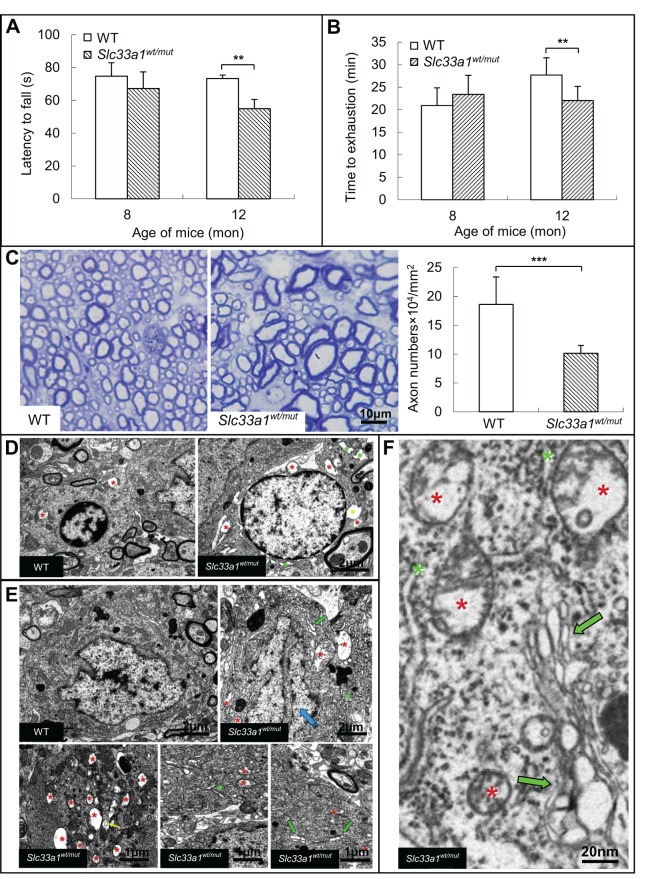
**Abnormal phynotypes of *Slc33a1^wt/mut^* mice.** (A,B) Impaired movements of *Slc33a1^wt/mut^* mice. *Slc33a1^wt/mut^* mice showed shorter latency to fall on a Rotarod (A) and shorter time to reach exhaustion following an exercise tolerance test (B) compared with WT littermates at 8 and 12 months old. *n*=6 mice for each experimental condition. (C) Semi-thin sections of the lateral column of lumber spinal cord stained with Toluidine Blue showed a morphological difference of nerves between WT and mutant mice, with a reduced number of axons in 12-month-old *Slc33a1^wt/mut^* mice. *n*=6 mice for each experimental condition. (D) Transmission electron micrographs of the gray matter of the lumber spinal cord from 12-month-old *Slc33a1^wt/mut^* mice showed swollen and degenerated mitochondria with cristae deficiency (red asterisks), abnormal endoplasmic reticulum (green asterisks), and perinuclear space expansion (yellow asterisks) in glial cells. (E,F) Transmission electron micrographs of the gray matter of the lumber spinal cord in 12-month-old *Slc33a1^wt/mut^* mice showed abnormal endoplasmic reticulum and increased free ribosome (green asterisks), swollen and degenerated mitochondria with cristae deficiency (red asterisks), swollen Golgi apparatus cisternae (green arrows), and nuclear pocket formation (blue arrow) in neurons. Data presented as mean±s.d.; **P<0.01, ***P<0.001 compared with WT group by *t*-test.

*Slc33a1^wt/mut^* mice bred normally and showed no overt phenotypic abnormalities when examined at 8 and 12 months old using a hind-leg clasping reflex test. All mice were able to hold their hind limbs apart with paw external rotation, indicating no apparent hindlimb locomotion deprivation (Fig. S1). Examination of muscular phenotype also revealed no significant difference between WT and *Slc33a1^wt/mut^* mice (Fig. S2). Congenital cataracts, hearing loss, and low serum copper and ceruloplasmin, phenotypes reported to be present in other SLC33A1 mutations (Schlipf et al., 2010; Huppke et al., 2012) were not detected in the *Slc33a1^wt/mut^* mice and individuals with SPG42 (Fig. S3). However, both rotarod and exercise tolerance tests showed that the 12-month-old *Slc33a1^wt/mut^* mice exhibited a decline in locomotion and significant hindlimb weakness compared with age-matched WT littermates (Fig. 3A,B).

### Central neurodegeneration in *Slc33a1^wt/mut^* mice

To examine the axonal degeneration in spinal cords of *Slc33a1^wt/mut^* mice, Luxol Fast Blue staining was performed, and the profound demyelinating phenotype observed in spinal cord white matter of *Slc33a1^wt/mut^* mice compared with that of the WT littermates (Fig. S4). Thus, we next determined, by Toluidine Blue staining, a significant reduction in the number of axons in lateral columns of lumber spinal cord of the 12-month-old *Slc33a1^wt/mut^* mice, and many axons were irregularly shaped when compared with WT littermates (Fig. 3C).

Transmission electron microscopy revealed degeneration within the gray matter of lumber spinal cord of 12-month-old *Slc33a1^wt/mut^* mice, such as swollen and degenerated mitochondria with cristae deficiency, abnormal endoplasmic reticulum, and perinuclear space expansion in glial cells of the spinal cord (Fig. 3D). In addition, abnormal endoplasmic reticulum, increase in free ribosomes, swollen and degenerated mitochondria with cristae deficiency, swollen Golgi apparatus cisternae, and nuclear pocket formation were evident in the neuronal cell body of spinal cord in *Slc33a1^wt/mut^* mice (Fig. 3E,F). In contrast, examination of sciatic nerves revealed no significant difference between WT and *Slc33a1^wt/mut^* mice (Fig. S5).

### Upregulation of the BMP signaling in *Slc33a1^wt/mut^* mice

**Fig. 4.**
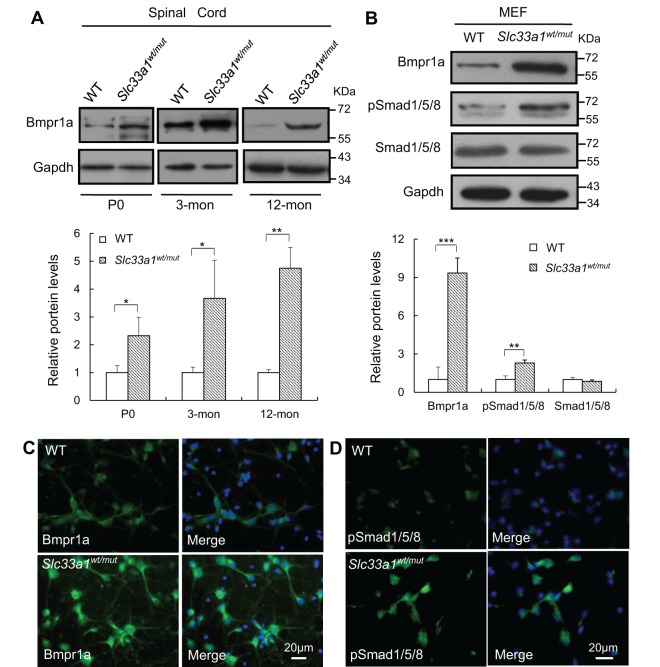
**Upregulated BMP signaling in *Slc33a1^wt/mut^* mice.** (A) Western blotting showed increased level of Bmpr1a in the spinal cord of *Slc33a1^wt/mut^* mice compared with WT littermates at P0, 3 months and 12 months old. The levels of Bmpr1a were quantified by densitometric analysis. *n*=6 mice each for experimental condition. (B) Western blotting showed increased level of Bmpr1a and pSmad1/5/8 in mouse embryonic fibroblasts (MEFs) of *Slc33a1^wt/mut^* mice compared with WT. The levels of Bmpr1a, pSmad1/5/8, and Smad1/5/8 were quantified by densitometric analysis. Data from three independent experiments. (C,D) Immunofluorescence staining of Bmpr1a (C) and pSmad1/5/8 (D) in primary cortical neurons from *Slc33a1^wt/mut^* mice compared with WT littermates. Data presented as mean±s.d.; *P<0.05, **P<0.01, ***P<0.001 compared with WT group by *t*-test.

We next assessed whether the SLC33A1^S113R^ mutant protein would impact BMP signaling. There was a significant increase in the amount of Bmpr1a protein in the spinal cords of 12-month-old *Slc33a1^wt/mut^* mice (Fig. 4A). Importantly, increased Bmpr1a levels were also detected in newborn and 3-month-old *Slc33a1^wt/mut^* mice. We were unable to detect the baseline levels of pSmad1/5/8 in spinal cords by western blotting analysis (data not shown). However, levels of pSmad1/5/8 and Bmpr1a were significantly increased in mouse embryonic fibroblasts and primary cultured cortical neurons derived from *Slc33a1^wt/mut^* mice (Fig. 4B-D).

### Injury-induced peripheral axonal regeneration was accelerated in *Slc33a1^wt/mut^* mice by upregulating BMP signaling

BMP signaling pathway has been reported to be hyperactivated during PNS injury (Tsujii et al., 2009). We therefore used a mouse sciatic nerve crush injury model to address the effect of *Slc33a1^S113R^* on the modulation of BMP signaling.

**Fig. 5.**
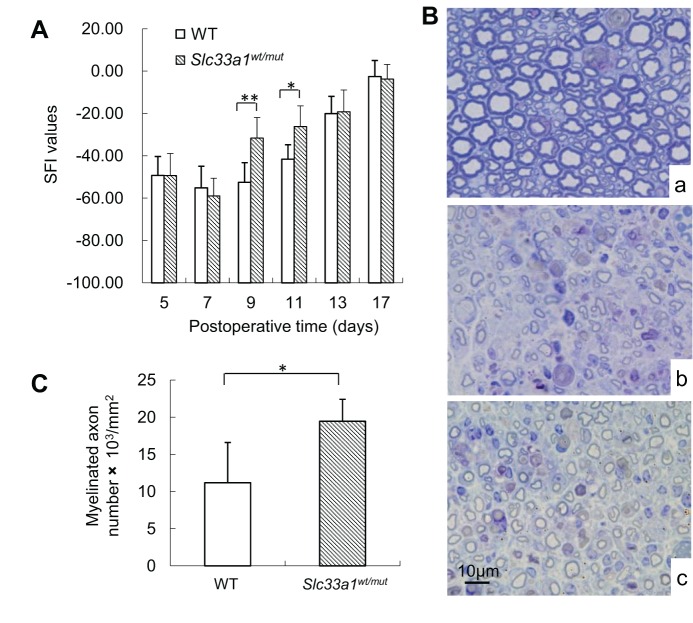
***Slc33a1^wt/mut^* mice have accelerated injury-induced sciatic nerve regeneration.** (A) Measurements made from walking track prints were then submitted to SFI. *n*=6 mice for each experimental condition. (B) Toluidine Blue staining of sciatic nerve transverse semi-thin sections. At week 2 after injury, many small-diameter axons with a thin myelin sheath were observed in the crushed sciatic nerve of WT (b) and *Slc33a1^wt/mut^* (c) mice at 5 mm distal to the injury site compared with the native nerve group (a). (C) Quantification of the number of myelinated axons. *n*=6 mice for each experimental condition. Data presented as mean±s.d.; *P<0.05, **P<0.01 compared with WT group by *t*-test.

The sciatic functional index (SFI) reflects functional nerve recovery. SFI values showed no significant differences between WT and *Slc33a1^wt/mut^* mice up to 7 day after crush injury. However, when examined 9 days and 11 days after crush injury, the SFI values in *Slc33a1^wt/mut^* mice were significantly higher than in WT littermates (Fig. 5A). Two weeks following crush injury, specimens of the crushed sciatic nerve at 5 mm distal to the injury sites were subjected to histological studies. As controls, tissue specimens from the contralateral uninjured sciatic nerve were also examined. Many small-diameter axons with a thin myelin sheath were observed in both groups (Fig. 5B). However, the number of myelinated axons in *Slc33a1^wt/mut^* mice was significantly higher than that in WT littermates (Fig. 5C). These results indicate that functional nerve recovery following crush injury was accelerated in *Slc33a1^wt/mut^* mice.

**Fig. 6.**
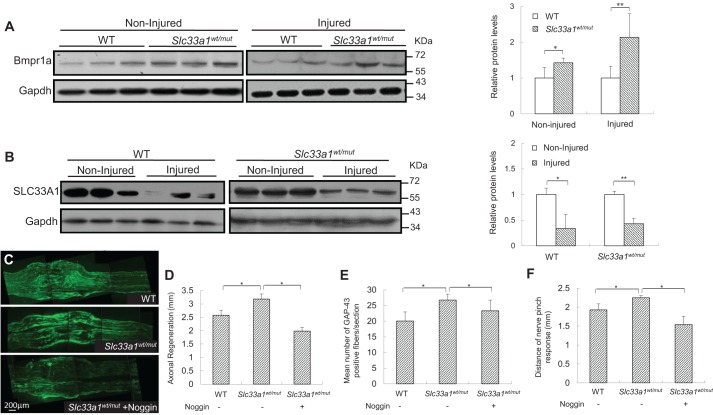
**Inhibition of BMP signaling with noggin attenuated accelerated injury-induced axonal regeneration in *Slc33a1^wt/mut^* mice.** (A) Left panels: western blotting showed increased level of Bmpr1a in the sciatic nerve in *Slc33a1^wt/mut^* mice compared with WT littermates. Each lane corresponds to a different animal. *n*=6 mice for each experimental condition. Right panel: levels of Bmpr1a were quantified by densitometric analysis. (B) Left panels: western blotting showed decreased level of SLC33A1 in the sciatic nerve following a crush injury compared with non-injured controls. Each lane corresponds to a different animal. Right panel: levels of SLC33A1 were quantified by densitometric analysis. *n*=6 mice for each experimental condition. (C) Immunofluorescence staining of GAP43 showing accelerated axonal growth at day 2 following sciatic nerve crush in *Slc33a1^wt/mut^* mice compared with WT littermates. Noggin injection attenuated the acceleration in *Slc33a1^wt/mut^* mice. (D) Quantification of the distances of axon regrowth of sciatic nerve following a crush injury as determined by GAP43 staining. *n*=6 mice for each experimental condition. (E) Quantification of the numbers of GAP43-positive fibers 1.5 mm distal to the crush site. *n*=6 mice each for experimental condition. (F) Functional regeneration assessed using the pinch test showed increased regeneration at day 2 post-injury in *Slc33a1^wt/mut^* mice compared with WT littermates. Noggin injection attenuated the acceleration in *Slc33a1^wt/mut^* mice. *n*=6 mice for each experimental condition. Data presented as mean±s.d.; *P<0.05. **P<0.01 compared with WT group by ANOVA.

We next tested whether the accelerated axonal regeneration following sciatic nerve injury in *Slc33a1^wt/mut^* mice was a result of increased BMP signaling. Indeed, the Bmpr1a level in the sciatic nerves of *Slc33a1^wt/mut^* mice was higher than that in WT littermates (Fig. 6A). Moreover, crush injury induced a further increase in Bmpr1a level from *Slc33a1^wt/mut^* mice (Fig. 6A). Consistent with the negative regulation of BMP signaling by SLC33A1, there was a significant decrease in the level of SLC33A1 protein in the sciatic nerves at day 2 after injury compared with non-injured controls (Fig. 6B). To determine whether inhibition of BMP signaling could attenuate the accelerated axonal regeneration following injury, we injected 1 µg of noggin into the sciatic nerve of *Slc33a1^wt/mut^* mice immediately prior to sciatic nerve crush. Histological analysis and functional measurement at day 2 after crush injury showed that noggin treatment efficiently attenuated the accelerated injury-induced axonal regeneration in *Slc33a1^wt/mut^* mice, as determined by GAP43 expression level and pinch test (Fig. 6C-F). These results suggest that accelerated injury-induced axonal regeneration in *Slc33a1^wt/mut^* mice is mediated through upregulation of BMP signaling.

### Accelerated axonal growth in DRG from *Slc33a1^wt/mut^* mice was attenuated by inhibition of BMP signaling

**Fig. 7.**
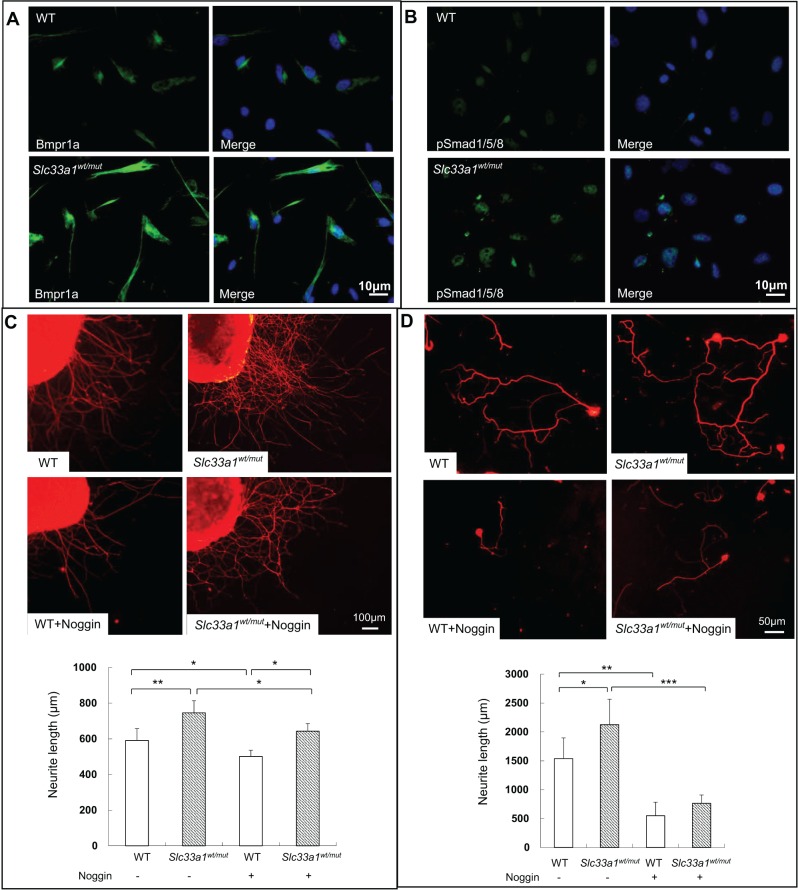
**Inhibition of BMP signaling with noggin attenuated hereditary spastic paraplegia in DRG from *Slc33a1^wt/mut^* mice.** (A,B) Immunofluorescence staining of Bmpr1a (A) and pSmad1/5/8 (B) in DRG neurons from *Slc33a1^wt/mut^* mice compared with WT littermates. (C) Upper panels: immunofluorescence staining of β-tubulin III in whole DRG explants from *Slc33a1^wt/mut^* and WT littermates after 48 h in culture. Noggin attenuated the accelerated neurite formation in DRG explants from *Slc33a1^wt/mut^* mice. Lower panel: quantification of the neurite length of DRG. *n*=6 mice each experimental condition. (D) Upper panels: immunofluorescence staining of β-tubulin III in DRG neurons from *Slc33a1^wt/mut^* and WT littermates after 48 h in culture. Noggin attenuated the accelerated neurite growth in DRG neurons from *Slc33a1^wt/mut^* mice. Lower panel: quantification of the neurite length of DRG. *n*=6 mice each experimental condition. Data presented as mean±s.d.; *P<0.05, **P<0.01, ***P<0.001 compared with WT group by ANOVA.

To further confirm the above results, we cultured neonatal whole DRG explants and dissociated DRG neurons from *Slc33a1^wt/mut^* mice and their WT littermates. Immunofluorescence analysis showed increased levels of Bmpr1a and pSmad1/5/8 in the DRG neurons from *Slc33a1^wt/mut^* mice (Fig. 7A,B). DRG neurons derived from *Slc33a1^wt/mut^* mice were able to extend more and much longer axons than adult naïve neurons in both cultured whole DRG explants and dissociated DRG neurons (Fig. 7C,D). Exposure to noggin attenuated the accelerated neurite growth in both DRG explants and dissociated DRG neurons from *Slc33a1^wt/mut^* mice (Fig. 7C,D).

To exclude a possible interfering effect of the neomycin cassette in the mutant allele (Fig. 1), we crossed *Slc33a1^S113R^* knock-in mice with Sox2-Cre mice to generate a new mutant allele without neomycin cassette. DRG explants and dissociated DRG neurons from the mice carrying the new allele behaved similarly to those from mice with the neomycin cassette (Fig. S6). Together, these data demonstrate that *Slc33a1* mutation enhances BMP-dependent neurite formation in DRG neurons *in vitro* and that noggin treatment can attenuate enhanced neurite outgrowth of DRG from *Slc33a1^wt/mut^* mice.

## DISCUSSION

Given that HSP is a length-dependent distal axonopathy of the corticospinal tract, and axons of the upper motor neuron in human are markedly longer than those in mouse, the locomotor impairment in mouse models of HSP is usually much milder than in humans with the same or equivalent mutations (Lo Giudice et al., 2014). In this study, we demonstrated that *Slc33a1^wt/mut^* mice with an S113R mutation exhibited HSP-related phenotypes, such as progressive impairments in motor function and axonal degeneration, which are milder than but similar to those in individuals with HSP carrying this mutation. We previously showed that knockdown of the *slc33a1* gene could result in scarce and poorly organized motor axons in zebrafish (Lin et al., 2008), which can be rescued by human wild-type mRNA, but not mutant RNA. Recently, Peng et al. (2014) reported another line of *Slc33a1^S113R^* knock-in mice that displayed neurodegeneration in both the central and peripheral nervous systems. These results together validated *SLC33A1^S113R^* mutation as a truly pathogenic mutation. However, whereas Peng et al. also observed early developmental arrest of homozygous mutants, they were able to detect *Slc33a1^mut/mut^* embryos at E10.5 and estimated that the arrest occurs at E8-8.5 based on the stage of the neural tube closure, which is in contrast to the more severe phenotypes in our line. Different strain backgrounds might have contributed to the different degree of abnormalities in the two lines of mutant mice.

Bone morphogenetic proteins (BMPs) have been implicated in many aspects of biological processes, including embryonic induction, pattern formation, cell proliferation, apoptosis and differentiation (Tsujii et al., 2009). Several lines of evidence suggest that BMP signaling is essential for orchestration of embryonic development and maintenance of tissue homeostasis in adult animals. Tight spatiotemporal control of BMP gradients plays a crucial role in controlling developmental patterning, such as dorsal-ventral patterning in neurodevelopment. Mice with null function of genes encoding BMPs, BMP receptors and their downstream signal transducers are usually embryonic lethal. Embryos lacking a functional *Bmpr1a* gene die at around the time of gastrulation without forming any mesoderm. It is therefore plausible that BMP signaling perturbation might play a role in the lethality of our *Slc33a1^mut/mut^* embryos.

Several HSP proteins, including atlastin-1 (also known as SPG3A), spastin (SPG4), NIPA1 (SPG6), spartin (SPG20) and PNPLA6, are associated with altered BMP signaling (Fassier et al., 2010; Song et al., 2013; Wang et al., 2007). NIPA1 inhibits BMP signaling by promoting endocytosis and lysosomal degradation of BMP receptors (Wang et al., 2007). Using a *Drosophila* model, Nahm et al. (2013) showed that Spartin inhibits neuronal BMP signaling by regulating the endocytic internalization and subsequent endosomal trafficking of the type II BMP receptor Wit, and that elevated BMP signaling contributes to neurodegeneration in *spartin* mutants. Our previous study showed that knockdown of *slc33a1* in zebrafish leads to upregulation of BMP signaling and impaired axonal outgrowth of motor neurons. Importantly, pharmacological blockade of BMPR1 activity by dorsomorphin can efficiently rescue the phenotypic defects in *slc33a1* knockdown zebrafish, which indicated that *Slc33a1^S113R^* mutation likely causes SPG42 via affecting BMP signaling (Mao et al., 2015). In this study, we show that BMP signaling is also elevated in the nervous system of *Slc33a1^S113R^* mutant mice. As in SPG42 patient-derived fibroblasts, Bmpr1a levels were increased in the nervous system and embryonic fibroblasts of *Slc33a1^S113R^* mutant mice. Our previous study suggested that the elevated BMP signaling caused by SLC33A1 mutation is likely the result of impaired degradation of BMPR1A. Future studies are needed to clarify the mechanism underlying SLC33A1-mediated BMPR1A degradation.

BMP signaling seems to have differential effects on neurite extension and axonal growth in the central and peripheral nervous systems. In rodents, BMP signaling is upregulated following lesion of the corticospinal tract, and suppression of this upregulation can promote regrowth of axons (Matsuura et al., 2008). BMP signaling is also upregulated after sciatic nerve injury, but diminished BMP signaling leads to retarded early axonal regeneration (Tsujii et al., 2009; Ma et al., 2011). *Slc33a1^wt/mut^* mice exhibited accelerated injury-induced peripheral axonal regeneration that was associated with elevated BMP signaling. Importantly, administration of BMP signaling antagonist could rescue accelerated axonal regrowth. These results suggest that elevated BMP signaling stimulates axonal regeneration in the PNS of *Slc33a1^wt/mut^* mice after injury. However, accelerated injury-induced peripheral nerve regeneration is unlikely to contribute to HSP, as neurodegeneration is the prominent feature of HSP. The mechanism by which elevated BMP signaling causes HSP clearly needs further investigation.

## MATERIALS AND METHODS

### Generation of *Slc33a1^S113R^* knock-in mice

*Slc33a1^S113R^* knock-in mice were generated at the National Resource Center of Mutant Mice/Model Animal Research Center of Nanjing University. All animal experiments were performed in compliance with national regulations and approved by the Animal Care and Use Committee, Shandong University, School of Medicine. The *S113R* mutation was introduced into a BAC clone containing the *Slc33a1* gene and flanking regions (RP24-179B11) using the *rpsL-neo* counter-selection method (supplied by the National Resource Center of Mutant Mice/Model Animal Research Center of Nanjing University). Then, a 10.5 kb DNA fragment, spanning the region from the 5′-flanking sequence to intron 1 of the *Slc33a1* gene, was recombined from the BAC clone into a vector pL253 which contained a TK cassette. A *loxP-neo-loxP* cassette was inserted into intron 1. Correctly targeted embryonic stem cell clones were confirmed by Southern blot analysis. The DNA fragments generated with *Eco*RV restriction enzyme were probed with the 5′ probe, and yielded a 12.9 kb fragment for the WT allele and a 8.0 kb fragment for the targeted allele. Analysis using *Nhe*I restriction enzyme with the 3′ probe revealed a 11.2 kb fragment for the WT allele and a 8.4 kb fragment for the targeted allele (Fig. 1A,B). Amino acid substitution in correctly targeted embryonic stem cells was confirmed by DNA sequencing. Chimeric mice derived from embryonic stem cells carrying the *Slc33a1^S113R^* knock-in mutation were crossed to WT mice to produce *Slc33a1^S113R^* knock-in C57BL/6J mice.

### Genotyping

Embryos at 18.5 days and neonates were genotyped by PCR or PCR-RFLP of total cellular DNA isolated from tissue. For the PCR-based genotyping, oligonucleotide primers (F1: *Slc33a1* upper, 5′-GATGTTTGTCCTTCTGCTTGC-3′; R1: *Slc33a1* lower, 5′-TCAGTAGATGGGTAAATGGG-3′; R2: *neo* lower, 5′-AAGGGTTATTGAATATGATCGGA-3′) were used concomitantly in a multiplex PCR to amplify corresponding *Slc33a1* alleles on mouse chromosome 3. Amplified products were separated on 1.5% agarose gels (Fig. 1C).

For the PCR-RFLP-based genotyping, oligonucleotide primers (F2: *Slc33a1* upper, 5′-CCCTGGTGACTTACCTAAAGC-3′; R3: *Slc33a1* lower, 5′-CCATCAATATTTCCGAGCAAACG-3′) were used. The PCR products were digested with *Hpa*II, and separated on 1.5% agarose gels (Fig. 1D).

Genotyping of the E2.5 and E3.5 embryos was performed by nested PCR. Individual embryos were lysed and subjected to PCR amplification using the following primers: for the first round – upper NEST-F1, 5′-TATGTCACCGACCATCTCCC-3′; lower NEST-R1, 5′-GAGGCTGAAACCGCAAGTA-3′; for the second round – upper NEST-F2, 5′-CCCTGGTGACTTACCTAAAGC-3′; lower NEST-R2, 5′-CCATCAATATTTCCGAGCAAACG-3′.

### E2.5 embryo culture *in vitro*

E2.5 embryos from heterozygote intercrosses were flushed out of the oviduct with M2 medium (Sigma), and were cultured for 3 days in complete ES medium (DMEM medium supplemented with 15% fetal bovine serum, 0.1 mM beta-mercaptoethanol, 4 mM glutamine, and 10^3^ units/ml recombinant leukemia inhibitory factor).

### Locomotion studies

#### Rotarod analysis

Motor performance was evaluated with a Rotarod apparatus. WT and *Slc33a1^wt/mut^* male mice were analyzed at 8 and 12 months of age (*n*=6 per group). Then the mice were placed on the rod accelerated linearly from 4 to 40 rpm over 5 min. All the mice were placed on the Rotarod apparatus for eight trials (four trials per day on two consecutive days) with a 60-min rest interval between trials. The time of hold on the rod was scored.

#### Exercise tolerance tests

WT and age-matched heterozygous male mice were analyzed at 8 and 12 months of age (*n*=6 per group). Each mouse was placed on the belt of a six-lane motorized treadmill supplied with shocker plates. The treadmill was run at 5 m/min for 5 min, followed by incremental increase of speed of 1 m/min every min until exhaustion, and the time to exhaustion was determined. Exhaustion was defined as when the mouse remained on the shocker plate for more than 20 s without attempting to re-engage the treadmill. Three tests were performed on the same animal, allowing 4 days between each test.

### Histological analysis

Mice were transcardially perfused with 4% paraformaldehyde, and the lumber spinal cord, mid-sciatic nerve and gastrocnemius were isolated and post-fixed in 4% paraformaldehyde. The transverse semi-thin sections (1 µm thick) from each sample were stained with Toluidine Blue. The number of myelinated axons was counted from at least six randomly selected fields under the magnification of ×400. Ultra-thin sections (70 nm thick) were observed under a transmission electron microscope (performed by Jinan Lujing Microscopic Technical Center). For the muscular phenotype, the gastrocnemius muscles were analyzed with multiple histochemical and histoenzymatic reactions including Hematoxylin and Eosin (H&E), cytochrome c oxidase (COX), succinate dehydrogenase (SDH), and nicotinamide adenine dinucleotide-tetrazolium reductase (NADH-TR) staining by standard methods (Oldfors et al., 2013).

### Western blotting

Protein samples were subjected to 12% SDS-PAGE gels and transferred to PVDF membranes (Amersham Pharmacia Biotech). After blocking, the membranes were incubated at 4°C overnight with antibodies against BMPR1A (1:200, Invitrogen, 38-6000), SLC33A1 (1:500, Abcam, ab83868), pSmad1/5/8 (1:400, CST, 13820), Smad1/5/8 (1:500, Abcam, ab66737), and GAPDH (1:5000, Sigma, SAB4300645). Proteins of interest were detected with horseradish peroxidase-conjugated secondary antibody (1:10,000, Jackson ImmunoResearch, 111-035-003) for 1 h at room temperature and visualized by ECL PLUS kit (Amersham Pharmacia Biotech). The results were quantified using ImageJ software (https://imagej.nih.gov/ij/).

### Primary cortical neurons culture

Primary cultures of cortical neurons were prepared from the cerebral cortices of E16 mice. Pregnant animals were killed by cervical dislocation and then fetuses were extracted. Neocortices were dissected with forceps, mechanically minced, and then trypsinized (0.05% trypsin-EDTA, Gibco) for 10 min at 37°C, gently triturated in fresh medium with 5% fetal bovine serum (Gibco), then seeded on glass coverslips pre-coated with 0.1 mg/ml poly-L-Lysine. Cortical neurons were cultured in Neurobasal Medium (Invitrogen) supplemented with 2% B-27 supplement (Gibco), 0.1 mg/ml L-glutamine (Sigma), then incubated at 37°C in a 5% CO_2_ incubator. After 3 days in culture, the cortical neurons were immunostained with antibodies against BMPR1A (1:200, Invitrogen, 38-6000), and pSmad1/5/8 (1:400, CST, 13820). Image analysis was carried out using ImageJ software.

### Sciatic nerve crushed model

Groups of WT mice and age-matched heterozygous mice weighting 25-30 g were anesthetized by an intraperitoneal injection of sodium pentobarbital (6 mg/100 g body weight, *n*=6 per group, half males and half females). The left sciatic nerve of mice was exposed through a gluteal muscle-splitting approach, and 1 μg noggin (1 μg/μl) or vehicle was injected directly into the sciatic nerve through a finely pulled glass micropipette. Subsequently sciatic nerves were crushed with smooth forceps for 30 s at the proximal thigh level.

### Sciatic nerve regeneration

On post-injury day 2 following sciatic crush, the mice were anesthetized to a level that pinching the skin of the contralateral uninjured right paw elicited a reflex withdrawal. The distance from the injury site to the most distal point on the nerve that produced a reflexive withdrawal when pinched was measured as the rate of regeneration.

GAP43 immunostaining was used to characterize regeneration at histology level. Sciatic nerves at the injury sites were sectioned, and the distances of injury-induced axon regrowth of sciatic nerve and the numbers of GAP43-positive fibers 1.5 mm distal to the crush site was determined.

Mouse walking track analysis was used to assess functional recovery following sciatic nerve injury. Footprints were recorded and analyzed before crush injury (0 week), and 5, 7, 9, 11 and 13 days following crush injury. The sciatic functional index (SFI) was calculated according to the following formula:
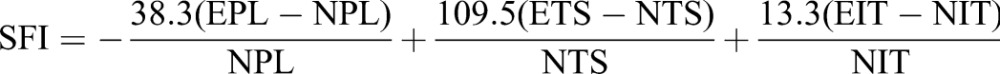
where EPL indicated the operated experimental paw length; NPL, normal paw length; ETS, operated experimental toe spread, i.e. the distance between the first and fifth toes; NTS, the normal toe spread; EIT, the operated experimental intermediary toe spread, i.e. the distance between the second and fourth toes; and NIT, normal intermediary toe spread. The SFI was scaled such that −100 represented the sciatic nerve was crushed completely and 0 represented normal function or completely recovery.

### DRG cultures

DRG explant cultures were prepared from the early postnatal day (P)0-P1 mouse. The L4 and L5 DRGs, supplying the sciatic nerve, were rapidly cleaned of spinal and peripheral roots and seeded on glass coverslips pre-coated with 2 μg/ml laminin. For the dissociated DRG neuron culture, the separated DRGs were trypsinized (0.05% trypsin-EDTA, Gibco) for 15 min at 37°C, gently triturated in fresh medium with 5% fetal bovine serum (Gibco), then seeded on the glass coverslips. DRGs were cultured in D-MEM/F-12 (1:1) (Invitrogen) supplemented with 5% fetal bovine serum (Gibco), 2% B-27 supplement (Gibco), 0.1 mg/ml L-glutamine (Sigma), with or without 2 μg/ml noggin, then incubated at 37°C in a 5% CO_2_ incubator. After 48 h in culture, the DRG were immunostained with antibodies against BMPR1A (1:200, Invitrogen, 38-6000), pSmad1/5/8 (1:400, CST, 13820), β-tubulin III (1:1000; Abcam, ab14545). Image analysis was carried out using ImageJ software.

### Statistical analysis

Data were presented as mean values±standard deviation. Data from two groups were evaluated statistically by two-tailed Student's *t*-test for any significant differences. Data were evaluated statistically by ANOVA to test for any differences among multiple groups. If significant differences were found by ANOVA, the Bonferroni method of multiple comparisons was used to determine which groups were significantly different from each other. A *P*-value of <0.05 was considered statistically significant.
